# Supplementary material for: Foliar Calcium Absorption by Tomato Plants: Comparing the Effects of Calcium Sources and Adjuvant Usage
Source: Plants (Basel). 2023 Jul 8;12(14):2587. doi: 10.3390/plants12142587 (PMC10385325; doi:10.3390/plants12142587)
Supplement: Supplementary file 1 [file plants-12-02587-s001.zip › plants-2467668-supplementary.pdf]

Supplementary Material

Foliar Calcium Absorption by Tomato Plants: Comparing the Effects of Calcium Sources and Adjuvant Usage

Eduardo Santos [1], Gabriel Sgarbiero Montanha [1,2], Luís Fernando Agostinho [3], Samira Polezi[3],
João Paulo Rodrigues Marques [1,4] and Hudson Wallace Pereira de Carvalho [1,*]

[1] Group of Specialty Fertilizers and Plant Nutrition, Laboratory of Nuclear Instrumentation, Centre for Nuclear Energy in Agriculture, University of São Paulo, Avenida Centenário, 303, Piracicaba 13400-970, Brazil; eduardosr07@usp.br (E.S.); gabriel.montanha@usp.br (G.S.M.); joaoanatomia@gmail.com (J.P.R.M.)
[2] Laboratory of Functional Genomics and Proteomics of Model Systems, Department of Biology and Biotechnology, Sapienza University of Rome, Via dei Sardi, 70, 00185 Rome, Italy
[3] Luiz de Queiroz College of Agriculture, University of São Paulo, Avenida Pádua Dias, 11, Piracicaba 13418-900, Brazil; lf.agostinho@usp.br (L.F.A.); sapolezi.silva@usp.br (S.P.)
[4] Department of Basic Science, Faculty of Animal Science and Food Engineering, University of São Paulo, Pirassununga 13635-900, Brazil
* Correspondence: hudson@cena.usp.br

Certificate Material Recovery

Satisfactory recovery values were achieved to Ca for both certificate material (105 and 96% for CRM-agro - c1005 and apple leaves- 1515, respectively), however, Sr recovery values were below 80% for CRM-agro - c1005 and 85% for apple leaves. Table S2 shows the concentration of Ca, Sr, P, K, Cu, Mg, Zn, and S.

Exponential decay function

Y0 refers to the moment when the plant reduces absorbing of the applied nutrient and reaches to a horizontal asymptote, **A1** is the initial fertilizer amount herein was normalized by the results of the first measurement as 100%, **e** represents a constant the Euler's number, **x** refers to elapsed time, and **t1** represents the probability of nutrient absorption. Through t1 value it is possible to calculate the absorption rate **(Abs. rate)** by equation S4. The time for plants absorbing 50% of what was applied on the leaf (**$t_{50\%}$**) was calculated by equation 3. Additionally, the amount of fertilizer absorbed when the plant reduces the kinetic of foliar absorption **(RAbs.)** can be measured by equation 4, which refers to the moment at which absorption curve reaches the horizontal asymptote.

Equation S1. $y=y0+A_1e^{-x/t1}$

Equation S2: $t_{50\%}= T1*\ln(2)$

Equation S3: **Abs.F= A1-y0**

Equation S4: **Abs. rate = 1/t1**

Table S1. Composition of foliar fertilizer used in the Line Scan analysis.

Treatment	Final Ca concentration (g L^{-1})	Final Sr concentration (g L^{-1})	Surfactant (%)
Ca/Sr Chloride	4.01	1.2	0
Ca/Sr Citrate	4.01	0.14	0
Ca/Sr Phosphate	4.01	0.15	0
Ca/Sr Chloride	4.01	1.2	1
Ca/Sr Citrate	4.01	0.14	1
Ca/Sr Phosphate	4.01	0.15	1

Table S2. The elementary composition of compounds was measured by ICP-OES.

**Compound**	**Ca**	**Sr**	**P**	**K**
		**g kg^{-1}**		
**Ca/Sr citrate**	134.9 ± 1.5	48.6 ± 0.2	-	9.66 ± 0.4
**Ca/Sr chloride**	4.0 ± 0.1	1.2 ± 0.0	-	-
**Ca/Sr phosphate**	16.0 ± 7.9	6.4 ± 3.1	8.4 ± 4.1	0.27 ± 0.2

**Compound**	**Cu**	**Mg**	**Zn**	**S**
		**mg kg^{-1}**		
**Ca/Sr citrate**	2.1 ± 1.2	5.1 ± 0.8	7.6 ± 2.1	36.6 ± 11.5
**Ca/Sr chloride**	-	2.0 ± 0	0.1 ± 0.0	1.9 ± 0.0
**Ca/Sr phosphate**	-	3.7 ± 1.1	-	29.6 ± 15.4

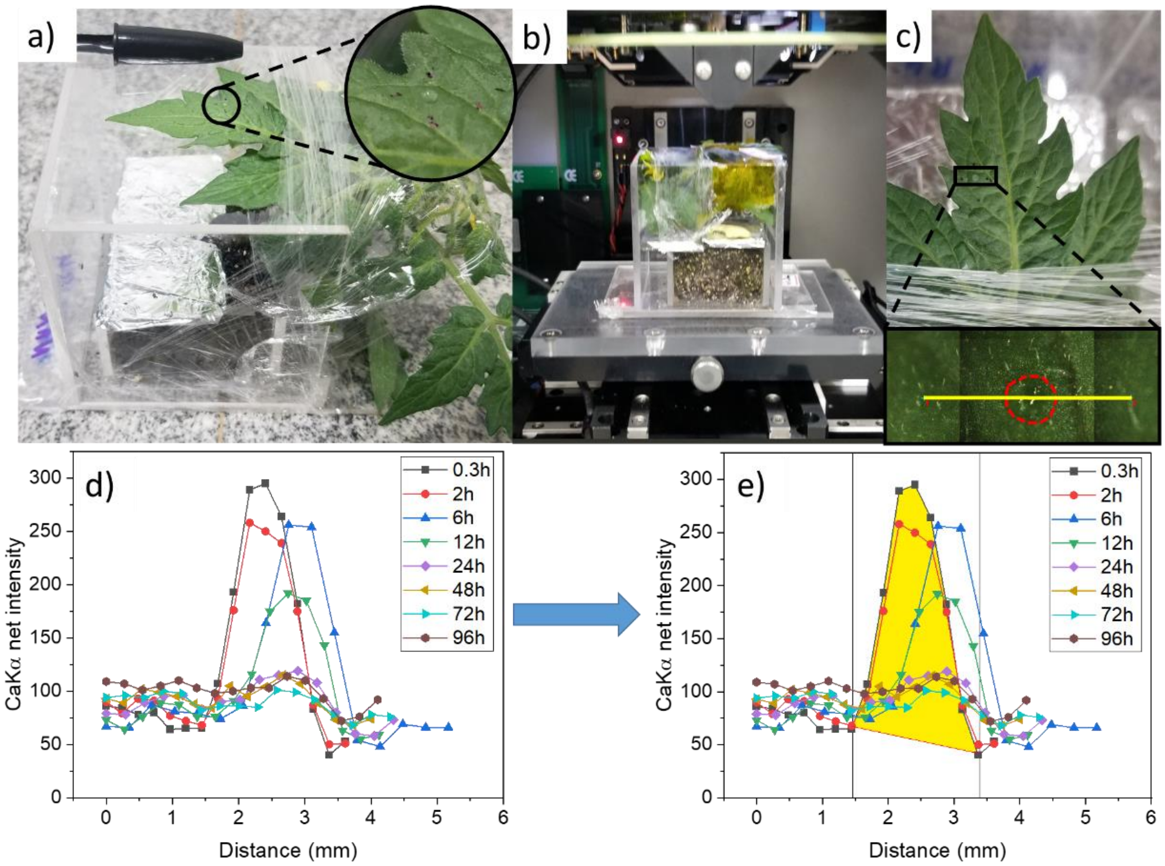

Figure S1. Method and results of previous sample characterization. Tomato plant in the acrylic sample holder detailing the drop fertilizer on the leaf (a). After dry time samples were analyzed by µ-XRF equipment. (b) In the line scan mode, the samples were analyzed for a period of 0.3h to 96h to monitor the fluctuation of Ca and Sr intensity using the 1 mm beam, 5s per point, and 16 points per line (c). Line scan results of calcium from 0.3h to 96h (d) integration of the area under the curve of Ca intensity (e).

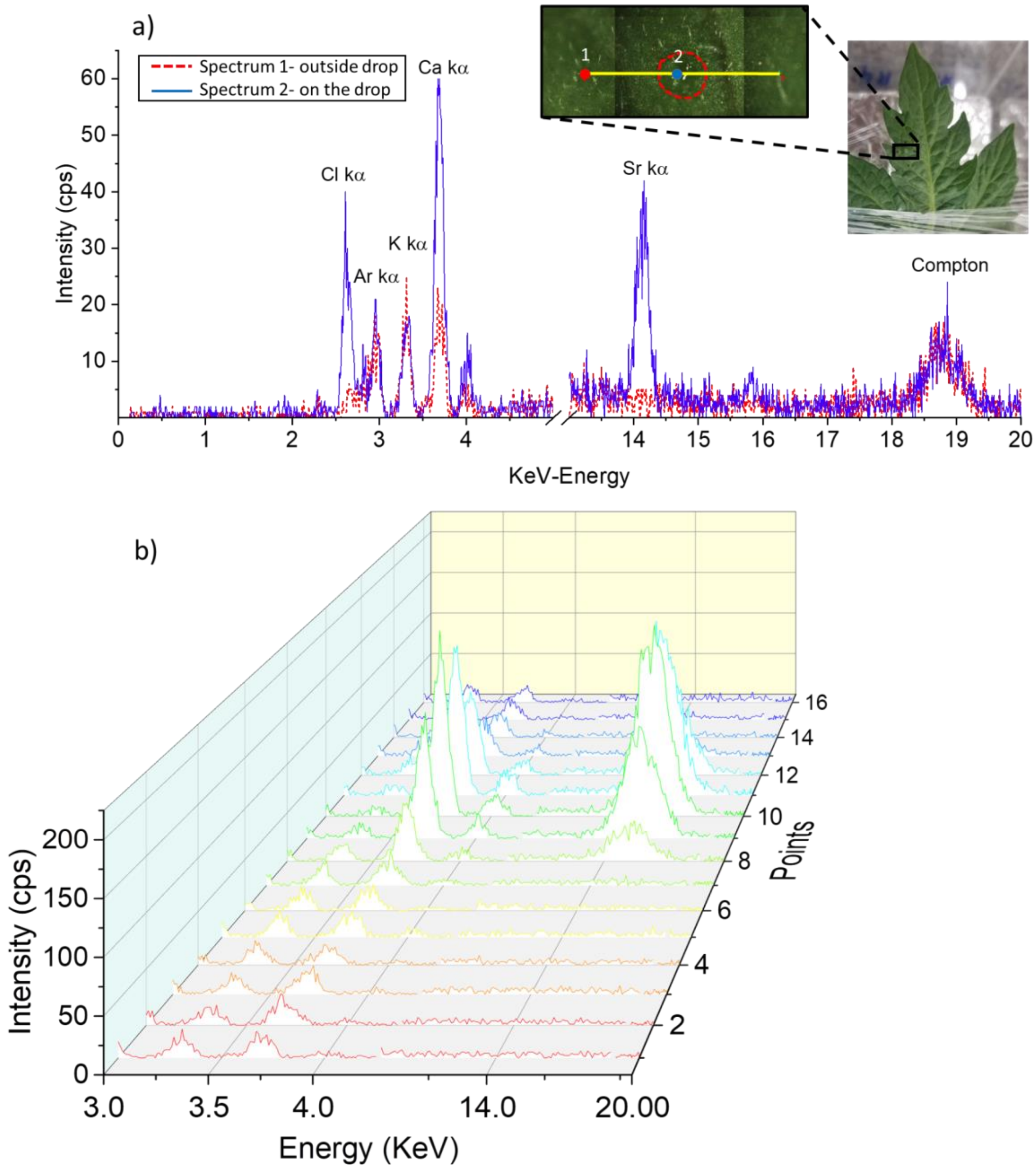

Figures S2. XRF spectra of Ca:Sr chloride to illustrate the 16-points line scan analysis. The spectrum of point 1 of line scan in red dash line and point 8 in blue continuous line a). All spectra of the 16-points line scan b). Note that the intensity of Ca and Sr do not appear in the spectra outside of the fertilizer drop, at the contrast spectrum on the drop of fertilizer intensity increase.

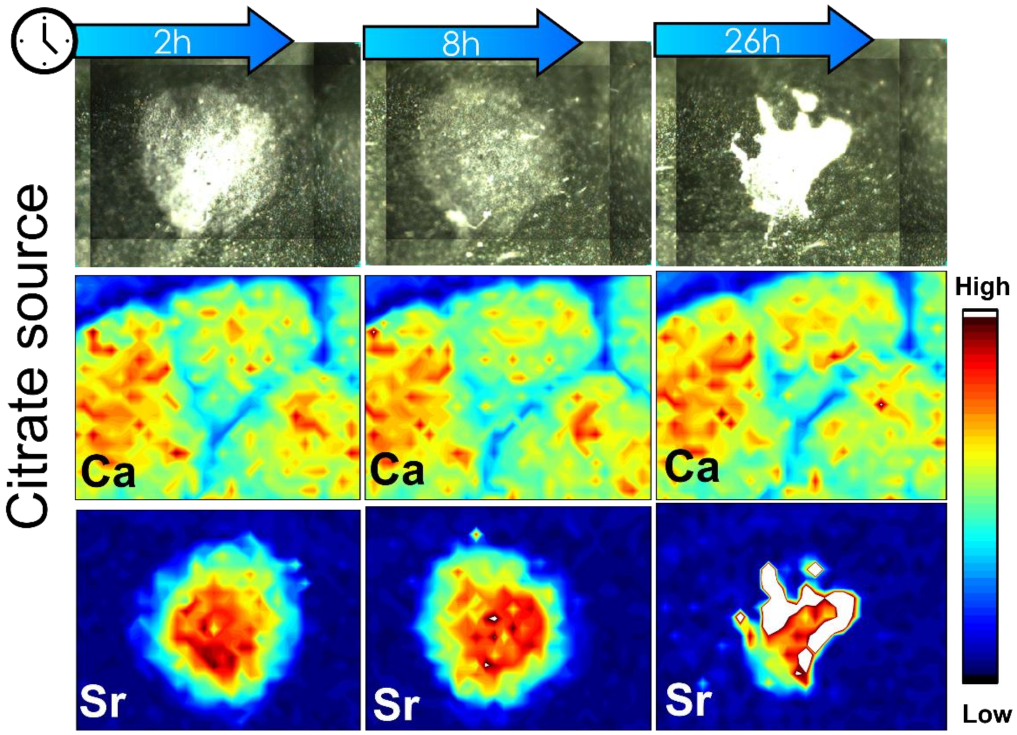

Figure S3. Photography of mapped area and its respective chemical map of Ca and Sr pass 2,8, and 26h of application. 0.5 uL of Sr citrate fertilizer was dropped on the leaf and three XRF analyses were measured in the same region. The experiment was conducted with 3 biological replicates that showed a similar profile.

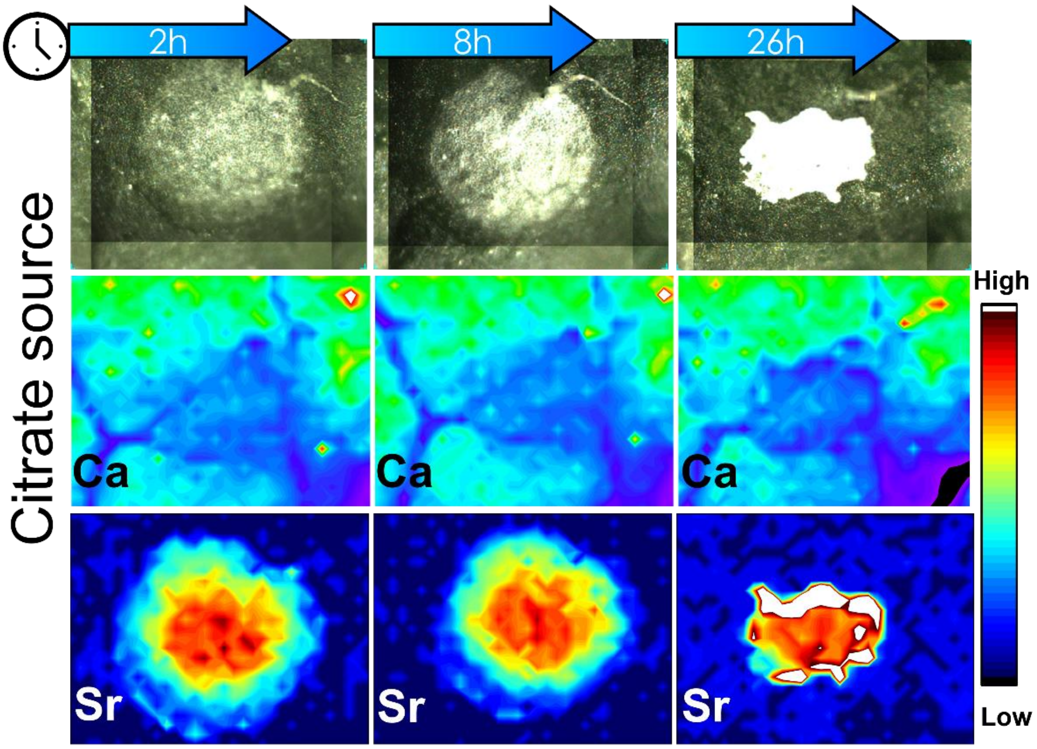

Figure S4. Photography of mapped area and its respective chemical map of Ca and Sr pass 2,8, and 26h of application. 0.5 uL of Sr citrate fertilizer was dropped on the leaf and three XRF

analyses were measured in the same region. The experiment was conducted with 3 biological replicates that showed a similar profile.

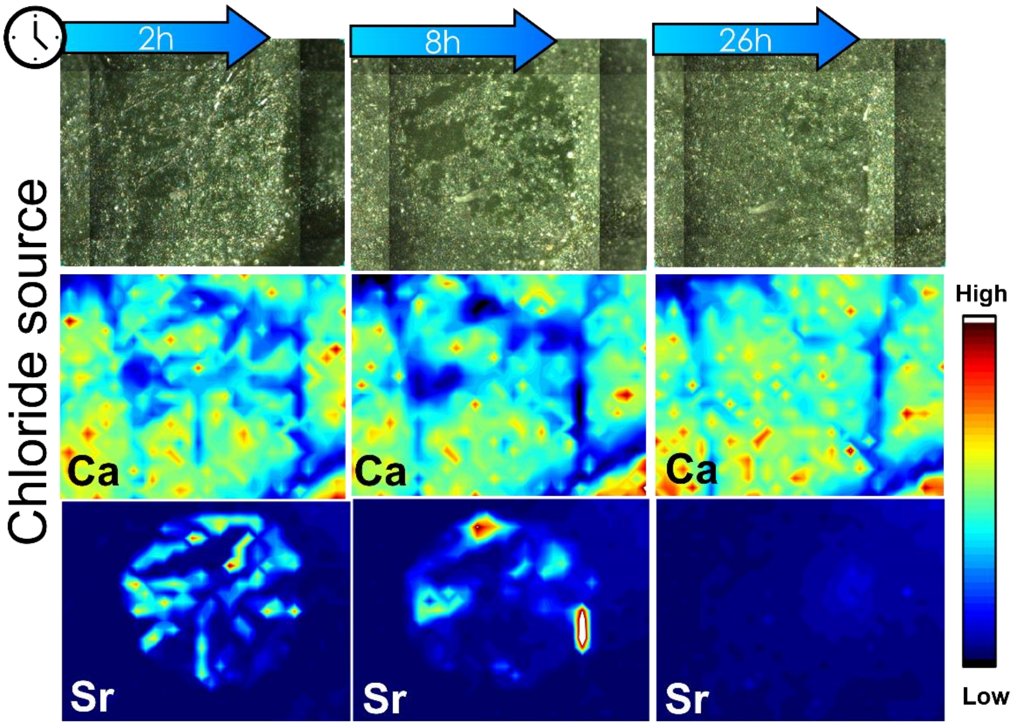

Figure S5. Photography of mapped area and its respective chemical map of Ca and Sr pass 2,8, and 26h of application. 0.5 uL of Sr chloride fertilizer was dropped on the leaf and three XRF analyses were measured in the same region. The experiment was conducted with 3 biological replicates that showed a similar profile.

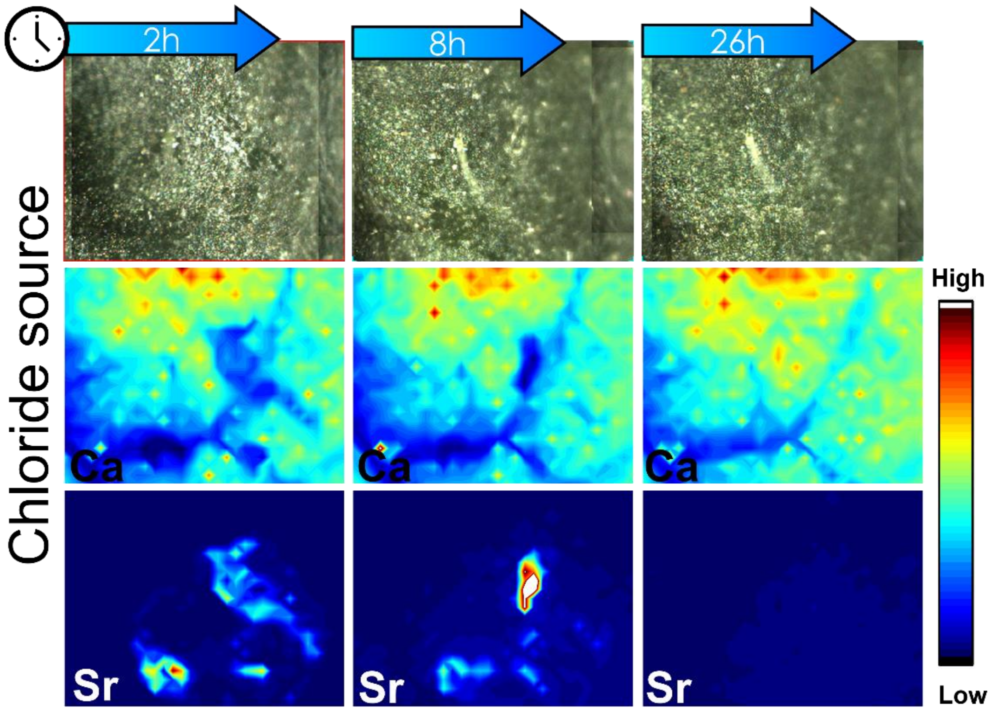

Figure S6. Photography of mapped area and its respective chemical map of Ca and Sr pass 2,8, and 26h of application. 0.5 uL of Sr chloride fertilizer was dropped on the leaf and three XRF

analyses were measured in the same region. The experiment was conducted with 3 biological replicates that showed a similar profile.
